# CaMKK2 facilitates Golgi-associated vesicle trafficking to sustain cancer cell proliferation

**DOI:** 10.1038/s41419-021-04335-x

**Published:** 2021-11-01

**Authors:** Lorna M. Stewart, Lisa Gerner, Mandy Rettel, Frank Stein, James F. Burrows, Ian G. Mills, Emma Evergren

**Affiliations:** 1 Patrick G Johnston Centre for Cancer Research, Queen’s University Belfast, 97 Lisburn Road, Belfast, BT9 7AE UK; 2 Nordic EMBL Partnership, Centre for Molecular Medicine Norway (NCMM), University of Oslo, P.O.Box 1137, Blindern, 0318 Oslo, Norway; 3 European Molecular Biology Laboratory (EMBL), Meyerhofstrasse 1, 69117 Heidelberg, Germany; 4 School of Pharmacy, Queen’s University Belfast, 97 Lisburn Road, Belfast, BT9 7BL UK; 5 Nuffield Department of Surgical Sciences, University of Oxford, John Radcliffe Hospital, Headley Way, OX3 9DU UK

**Keywords:** Prostate cancer, Golgi

## Abstract

Calcium/calmodulin-dependent protein kinase kinase 2 (CaMKK2) regulates cell and whole-body metabolism and supports tumorigenesis. The cellular impacts of perturbing CAMKK2 expression are, however, not yet fully characterised. By knocking down CAMKK2 levels, we have identified a number of significant subcellular changes indicative of perturbations in vesicle trafficking within the endomembrane compartment. To determine how they might contribute to effects on cell proliferation, we have used proteomics to identify Gemin4 as a direct interactor, capable of binding CAMKK2 and COPI subunits. Prompted by this, we confirmed that CAMKK2 knockdown leads to concomitant and significant reductions in δ-COP protein. Using imaging, we show that CAMKK2 knockdown leads to Golgi expansion, the induction of ER stress, abortive autophagy and impaired lysosomal acidification. All are phenotypes of COPI depletion. Based on our findings, we hypothesise that CAMKK2 sustains cell proliferation in large part through effects on organelle integrity and membrane trafficking.

## Introduction

Calcium/calmodulin-dependent protein kinase kinase 2 (CaMKK2) belongs to the serine/threonine-specific protein kinase family. It is activated by binding calcium–calmodulin (Ca^2+/^CaM) resulting in downstream activation of kinases CaMKI, CaMKIV and AMPK (α subunit of AMP-activated protein kinase) [1]. CaMKK2 is an important component of multiple signalling pathways that regulate glucose metabolism, adipogenesis, nutrient intake, inflammation and bone homoeostasis and has been shown to be deregulated in a number of diseases, such as obesity and cancer [2]. CAMKK2 is overexpressed in liver and prostate cancer [3–5]. In prostate cancer, its overexpression is in part due to androgen receptor (AR) activity [4]. Furthermore, inhibition and knockdown of CAMKK2 impaired androgen-responsive cell growth of prostate cancer cells [4]. Subsequently in vivo studies have shown that knocking out CAMKK2 in a transgenic model of prostate cancer driven by phosphatase and tensin homologue (PTEN) loss impairs tumorigenesis [6].

Biologically CAMKK2 has been implicated in the regulation of a range of subcellular processes including autophagy, vesicle trafficking [7–9], the regulation of actin dynamics [10], maintenance of cell–cell contacts and cell migration [11]. The cytoprotective role of autophagy in cancer to maintain membrane homoeostasis, manage endoplasmic reticulum (ER) stress and supply nutrients is well established [12]. In mapping the protein–protein interaction network for autophagy, CaMKK2 was found to co-immunoprecipitate not only proteins involved in autophagy [7]. Additional membrane trafficking proteins were identified as interactors in this study. The role of membrane trafficking in cancer biology is important because impaired endocytosis not only leads to trapping of growth factor receptors at the cell surface [13] and amplified cell signalling but also deregulation of Golgi-to-ER trafficking [14, 15] and vesicle trafficking [16, 17] affecting localisation, secretion and degradation of important proteins. Thus, the balance of intracellular vesicle trafficking is fundamental for processes critical for tumorigenesis such as proliferation, migration, invasion, exosome secretion and metabolism. It is therefore important to determine which biological processes are affected by CAMKK2 to explain its role in promoting the proliferation of cancer cells.

To address this important question, we set out to identify novel interaction partners using proteomics and we identified a protein complex that is associated with CaMKK2, containing Gemin4 and δ-COP. CAMKK2 expression was required to maintain δ-COP levels in cells and reducing CAMKK2 expression phenocopied known defects arising from dysregulated COPI coatomer complex function, including Golgi network expansion, impaired lysosomal acidification, abortive autophagy and reduced cell proliferation. Our data show that CAMKK2 sustain cell proliferation by regulating organelle integrity and homoeostasis.

## Materials and methods

### Reagents and antibodies

The following primary antibodies were used in this study: Gemin4 (rabbit, Cat# ARP56757_P050, Aviva Systems Biology and rabbit, Cat# HPA065699, Atlas antibodies); Gemin3 (mouse, Cat# 10305, AbCam); SMN1 (mouse, Cat# NB100-1936, Novus Biologicals); CaMKK2 (rabbit, Cat# HPA017389, Atlas antibodies); GAPDH (rabbit, Cat# 2118S, CST); ARCN1/delta-COP (rabbit, Cat# 96725, AbCam); alpha-COP (rabbit, Cat# HPA028024, Atlas Antibodies); zeta1-COP (rabbit, Cat# ARP70053, Aviva Systems Biology), Calnexin (rabbit, Cat# 22595, AbCam); actin (mouse, Cat# 8226, AbCam), TGN46 (sheep, Cat# AHP500GT, Bio-Rad); Cathepsin D (rabbit, Cat# ab75852, AbCam); IRE1α (rabbit, Cat# 3294S, CST); PERK (rabbit, Cat# 5683S, CST); CHOP (mouse, Cat# 2895S, CST); and p62 (rabbit, Cat# 5114, CST and mouse, Cat# 56416, AbCam).

The secondary antibodies used for immunocytochemistry were Alexa Fluor 488, 568, 594 goat anti-rabbit, anti-mouse and anti-sheep (Thermo Fisher Scientific); for western blot detection goat anti-rabbit and anti-mouse IgG-horseradish peroxidase (HRP) conjugates (Cell Signaling Technologies) and anti-glutathione *S*-transferase (GST)-HRP (Cat# Tap-0005, 2B Scientific).

The following gene expression constructs were used: pET28a-CaMKK2 aa 1–501 using restriction sites *Bam*HI and *Xho*I, pCi-CaMKK2-EGFP (NM_153499), pET32a-Gemin4 aa 1–370 and pEGFP Gemin4 (provided by Dr. Philip Young and Dr. Robert Morse, Peninsula Medical School, University of Exeter, UK) [18], and pGEX6p-Gemin4 aa 1–370 (subcloned from the pET32a clone using restriction sites *Eco*RI and *Xho*I). The short hairpin RNA (shRNA) constructs for *CAMKK2* were obtained from the NKI library in pRETROSUPER vector: shControl (5’-ATTACTGCCTTTGGCCTCG-3’), shCAMKK2-1 (5’- CTTAAGAGACAACTAAGCC-3’), and shCAMKK2-2 (5’GGGCTTGAAATTTAATAAG-3’); and the pEGFP-C-shRNA constructs for *GEMIN4* from Origene (Cat# TL304365V, Origene).

### Cell culture

All cell lines were obtained from ATCC. LNCaP cell culture media consisted of Roswell Park Memorial Institute 1640 (RPMI 1640, Gibco) supplemented with 10% foetal bovine serum (FBS, Gibco) or 10% charcoal-stripped FBS (CSS; Sigma-Aldrich) and 1% penicillin/streptomycin (Gibco). Phoenix and HEK293 cells were cultured in Dulbecco’s Modified Eagle Medium (Gibco) supplemented with 10% FBS and 1% penicillin/streptomycin. Cell lines were routinely tested for mycoplasma contamination (MycoAlert, Lonza). Polyethylenimine MAX (#24765, Polysciences) was used for transient transfections.

### Retroviral transductions

shRNA vectors were used to generate retrovirus [19]. LNCaP cells were infected with retrovirus and 24 h post infection 1.25 µg/ml puromycin (Sigma-Aldrich) was added for selection.

### Apoptosis and proliferation assays

To assess cell death, FITC Annexin V Apoptosis Detection Kit (BD Biosciences) was used according to the manufacturer’s instructions and analysed on a BD LSRII flow cytometer. Cell viability was assessed using Reazurin ‘Alamar Blue’ assay (Sigma-Aldrich) or CellTiter-Glo 2.0 (Promega) on a FluoStar Omega microplate reader.

### Protein expression and purification

pGEX6P-1 constructs for CaMKK2 isoform 2 and Gemin4 (aa 1–370) were expressed and purified as previously described [20].

### Peptide array

Peptide arrays were constructed on nitrocellulose membranes using MultiPep automated peptide synthesiser (Intavis Bioanalytical Instruments) as described [21]. The array was incubated with recombinant protein (1 µg/ml) in TBS-T for 2 h, washed and detected with GST-HRP-conjugated antibody.

### Avidin pulldown

Peptide pulldown was performed using the Pierce^TM^ Biotinylated Protein Interaction Kit (Thermo Scientific) according to the manufacturers’ instructions with LNCaP cell lysate grown in steady-state conditions or following 24 h 10 nM R1881 treatment or ethanol control. Two biotinylated peptides (GenScript, NJ, USA) were synthesised, the Gemin4 CaMKK2 Binding Motif (SLTSFSQNA) and a control peptide (LQPHPVTPS).

### Immunoprecipitation (IP)

LNCaP cells treated with 10 nM R1881 for 24 h, untreated or HEK293 cells expressing CaMKK2-GFP) were resuspended in IP Lysis Buffer (10 mM Tris-HCl pH8, 1 mM EDTA, 0.5 mM EGTA, 1% Triton X-100, 0.1% Na-deoxycholate, 0.1% LDS, 140 mM NaCl, cOmplete protease inhibitor cocktail) and lysed for 15 min at 4 °C. The protein extract was precleared with unspecific antibody (sc-2027, Santa Cruz) and Protein A Dynabeads (Invitrogen). For the endogenous IP of CaMKK2, 500 µg pre-cleared cell lysate was used per sample and was incubated with 2.5 µg CaMKK2 antibody or rabbit IgG overnight at 4 °C. Protein A Dynabeads were added to each sample before washing the beads and eluting proteins with sample buffer.

### Bioinformatics analysis

Mass spectrometry (MS) was performed to identify novel interaction partners from CaMKK2 IP as described by Bollineni et al. [22]. Data analysis followed the same method. To generate lists of proteins interacting with CaMKK2, Scaffold version 4.4 was used to validate MS/MS-based peptide/protein identification. To be identified, the protein had to have at least one unique peptide present in CaMKK2 IP and absent in IgG control.

MS and data analysis of the peptide pulldown was performed as described by Perez-Perri et al. [23] using TMT6plex labelling. Only proteins that had at least two unique peptides in at least two replicates were included. Proteins that are frequently co-purified in avidin pulldown experiments were excluded. Pathway analysis (https://maayanlab.cloud/Enrichr/; [24]) was performed on the remaining 883 proteins, and the enriched cellular components with an adjusted *p* value <0.001 were ranked based on the odds ratio.

### Immunofluorescence

Cells grown on glass coverslips were stained and imaged on an inverted Leica SP8 confocal microscope with a ×63 objective (1.4 NA HCX PL APO lens) as described [25]. At least 20 cells were randomly chosen using DAPI staining and analysed per condition.

### Live cell imaging

For multiphoton microscopy, cells were stained with Lysosensor DND-160 (Thermo Fisher Scientific) and imaged within 12 min using a Leica-TCS SP8 Multiphoton microscope set at 760 nm double photon excitation with a ×25/0.95NA HC FLUOSTAR water immersion lens. Separate channels were used to collect emitted fluorescence, corrected total cell fluorescence was calculated and a ratio between the blue and green channels generated to estimate lysosomal acidity.

### Transmission electron microscopy

Samples were prepared using a standard protocol [25]. Ultrathin sections were imaged on a Jeol JEM1400plus microscope at 80 kV equipped with a JEOL Ruby (8 MPixel) bottom-mounted CCD camera.

### Statistical data analysis

Statistical data analysis was performed using Prism version 8.0.0 (GraphPad Software) from at least three independent experiments, unless otherwise specified. For all quantifications provided, the means and SD are shown. The number of experimental and technical replicates are detailed in the figure legends. Outliers were identified by a ROUT test. Unless otherwise stated, statistical data analysis was performed with unpaired, two-tailed Student’s *t* test for comparison of data with a Gaussian distribution and equal variance. *P < 0.05; **P < 0.01; ***P < 0.001; ****P < 0.0001.

## Results

### Identification of novel CaMKK2 interaction partners

**Fig. 1:**
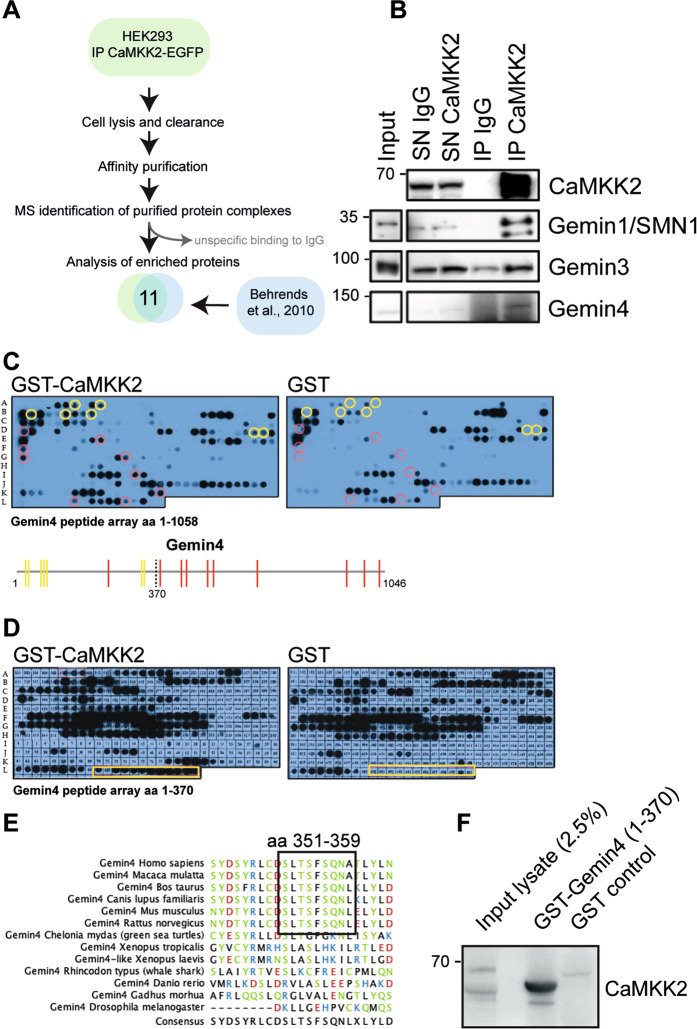
Identification of novel CaMKK2 interaction partners. **A** Schematic outlining the proteomics experimental design and workflow to identify CaMKK2-interacting proteins by co-immunoprecipitation (co-IP). HEK293 cells were transfected with EGFP-tagged CaMKK2, co-immunoprecipitated proteins were identified by mass spectrometry and the data were compared with a previously published data set [6]. **B** Immunoprecipitation of endogenous CaMKK2 from LNCaP cells showed co-immunoprecipitation of the Gemin complex. Immunoprecipitates (IP) with a control IgG were compared with those of CaMKK2 IgG, input lysate and unbound supernatant (SN). Bound proteins were evaluated by western blotting with the indicated antibodies. **C** A peptide array of full-length Gemin4 divided into 20 amino acid peptides with a 17 amino acid overlap was spotted on two nitrocellulose membranes. The membranes were incubated with either GST-CaMKK2 or GST alone. Bound GST protein was detected with a HRP-conjugated GST antibody. Yellow circles indicate peptides that bound CaMKK2 and had overlapping sequence with neighbouring peptides and red circles denote peptides that bound CaMKK2 but did not contain sequence overlap. A schematic drawing illustrates the sequence location of the highlighted peptides, which were concentrated in the first 370 aa of the Gemin4 sequence. **D** A peptide array of the N-terminal region of Gemin4 (aa 1–370) using 20 aa peptides with a 19 aa overlap. The peptide array was incubated with GST-CaMKK2 and GST control. The yellow box highlights the region in Gemin4 where GST-CaMKK2 but not GST bound. **E** Multiple sequence alignment of the CaMKK2-binding motif (CBM; aa 351–359) in Gemin4 identified in the peptide array illustrates sequence conservation across species. **F** To verify the specificity of the interaction in solution recombinant GST-tagged Gemin4 (aa 1–370) was used in a pulldown assay with LNCaP cell extracts. Equal amounts of control GST and GST-Gemin4 were used in the assay. The CaMKK2 binding was detected by western blotting.

Only one study has previously identified CaMKK2-interacting proteins using immuno-enrichment and mass spectroscopy [7]. This was undertaken in the HEK293 cell line and consequently we initially performed a comparable study in the same cell line by overexpressing CAMKK2. In so doing, 42% of the proteins that we identified overlapped with those that were identified in the earlier study (Fig. 1A). Of those, none were validated in the original study [7]. We have previously shown that CAMKK2 is an AR target gene and that its expression is induced by androgen treatment and maintained in recurrent disease following androgen-deprivation therapy [2]. To further determine which proteins and protein complexes may be most relevant for further functional studies, we performed endogenous CAMKK2 IP on protein extracts from the LNCaP cell line in the presence and absence of the synthetic androgen R1881. We identified 90 proteins in the androgen condition and 38 in the vehicle control condition potentially reflecting the higher levels of CAMKK2 expression upon androgen treatment (Supplementary Table 1). The only proteins that we identified in all conditions were known Gemin4 interactors, specifically Gemin1/SMN1 and Gemin3/DDX20. We confirmed these interactions with CAMKK2 by blotting immunoprecipitates for Gemin proteins (Fig. 1B) and by IPs of Gemin/SMN-1, -3, -4 (Supplementary Fig. 1). Gemin4 was included as it is a core protein in the Gemin complex and was previously identified in a proteomics study as a CaMKK2 interactor [7]. CaMKK2 co-IP was only detected in the Gemin4 sample suggesting that Gemin3 and Gemin1/SMN1 interact indirectly (Supplementary Fig. 1). Furthermore, Gemin4 is known to be regulated by the AR and associates with prostate cancer progression [4, 26], which is highly relevant in the context of prostate cancer and CaMKK2 biology as the AR is also a major driver of CaMKK2 expression, disease progression and biology [2]. Because our cell lines are prostate cancer derived, we chose to explore the interaction between Gemin4 and CaMKK2 further.

### CaMKK2 interacts directly with Gemin4 via a conserved motif in its N-terminal domain

To map the binding region of CaMKK2 in Gemin4, we made a peptide array for full-length Gemin4 consisting of 20-amino acid peptides with an overlap of 17 residues between each peptide spot. Due to the overlap of the peptides, a true binding site would appear as a positive signal in multiple peptide spots. The peptide array was incubated with GST-CaMKK2 protein or GST alone as a negative control. The strongest interaction was observed in the N-terminal region of Gemin4 where GST-CaMKK2 bound to multiple overlapping peptides within the first 371 amino acids (Fig. 1C). To further map the protein interaction, a second peptide array using this region of Gemin4 was designed consisting of 20 amino acids with a 19-amino acid overlap between each peptide spot. A continuous sequence of peptide spots covering amino acids 340–370 produced a signal with GST-CAMKK2 but not in the control condition (Fig. 1D). The consensus sequence present in all of these peptide spots consisted of 9 amino acids (‘SLTSFSQNA’ in the single-letter amino acid code), which are conserved in Gemin4 in mammals (Fig. 1E). We have termed this the CaMKK2-binding motif (CBM). Next, recombinant GST-tagged Gemin4 (amino acids 1–370) was expressed and incubated with LNCaP cell lysate. CAMKK2 was enriched in this pulldown assay compared to a GST control as determined by western blotting (Fig. 1F).

### The CBM in Gemin4 interacts with components of the COPI complex

**Fig. 2:**
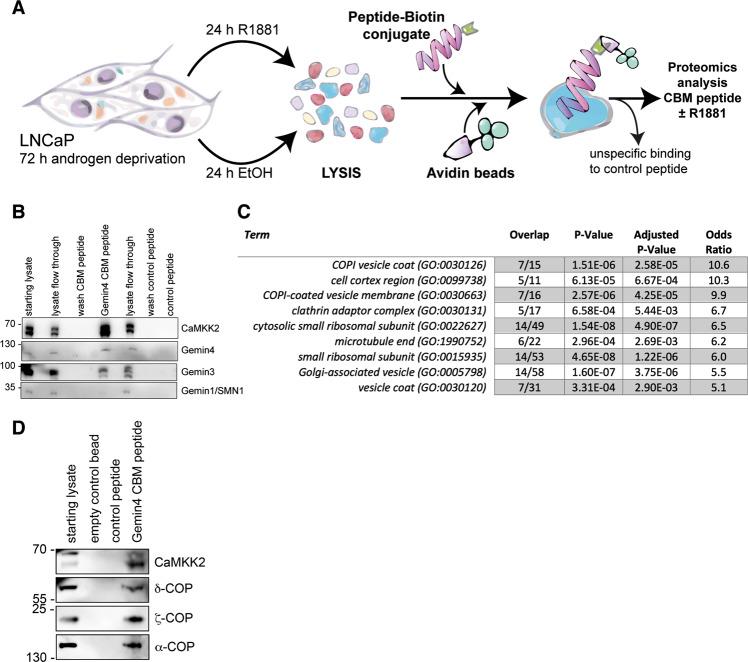
Identification of the COPI coat complex as a novel interaction partner of the CaMKK2-Gemin4 protein complex. **A** A schematic outlining the Gemin4 peptide pulldown experiment using biotinylated CaMKK2-binding motif (CBM) as bait from cell lysates of LNCaP cells treated with androgen (R1881) or an ethanol control. Bound proteins were identified by western blotting or mass spectrometry. **B** The streptavidin pulldown of biotinylated Gemin4 CBM and control peptide was analysed by western blotting to demonstrate enrichment of known interactors CaMKK2 and Gemin proteins. **C** Proteins that bound specifically to the Gemin4 CBM peptide were identified with mass spectrometry and the data set was assessed by pathway analysis (GO cellular location). The top ranked pathways are shown in a table. **D** A Gemin4 CBM peptide pulldown was used to validate the novel interactions of Gemin4 identified by the pathway analysis. Proteins bound by the peptide from LNCaP cell extract was analysed by western blot using antibodies for CaMKK2 and three components of the COPI complex; α-COP, δ-COP and ζ-COP.

To determine the extent to which this motif could recruit other known or novel Gemin4 interactors, we used it as biotinylated bait. We incubated this bait peptide with lysate from vehicle and androgen-treated cells and employed mass spectroscopy to identify bound proteins (Fig. 2A). A length-matched biotinylated peptide derived from a region of the N-terminus of Gemin4 that did not produce a binding signal for CAMKK2 in the peptide array experiment was used as a control (amino acids 78–86). Importantly, the Gemin4 CBM peptide showed a prominent enrichment of CaMKK2 compared to the cell lysate and also binding of Gemin4 and Gemin3 (Fig. 2B). There was no detectable binding to the control peptide. Another interactor that we had identified in the immunoprecipitates, Gemin1/SMN1, was not enriched by the CBM peptide indicating that this is a specific binding sequence (Fig. 2B). All of the proteins that were enriched by the CBM peptide and identified were subjected to a gene ontology enrichment analysis which showed that the majority of the ten most significant gene ontologies were related to Golgi and ER vesicle trafficking (Fig. 2C and Supplementary Table 2). COPI vesicle coat was the most significant ontology in the data set and specifically the COPI F-complex, an adaptor-like complex composed of the tetrameric β-, γ-, δ- and ζ-COP proteins, was found [27] (Supplementary Table 2).

Whereas previous studies have found α-, β- and γ-COP to be associated with the Gemin/SMN complex, δ-COP was novel and we took this forward for validation [28, 29]. The Gemin4 CBM peptide pulldown was probed for δ-COP, which showed a clear enrichment compared to the control peptide together with α-COP, ζ-COP and CaMKK2 (Fig. 2D). δ-COP was also found in CaMKK2 IPs (Supplementary Table 1; [7]), suggesting that it is an interaction partner of both CAMKK2 and Gemin4.

### Stable knockdown of CaMKK2 expression in LNCaP cells inhibits proliferation, COPI coatomer expression and deregulates Golgi apparatus morphology

**Fig. 3:**
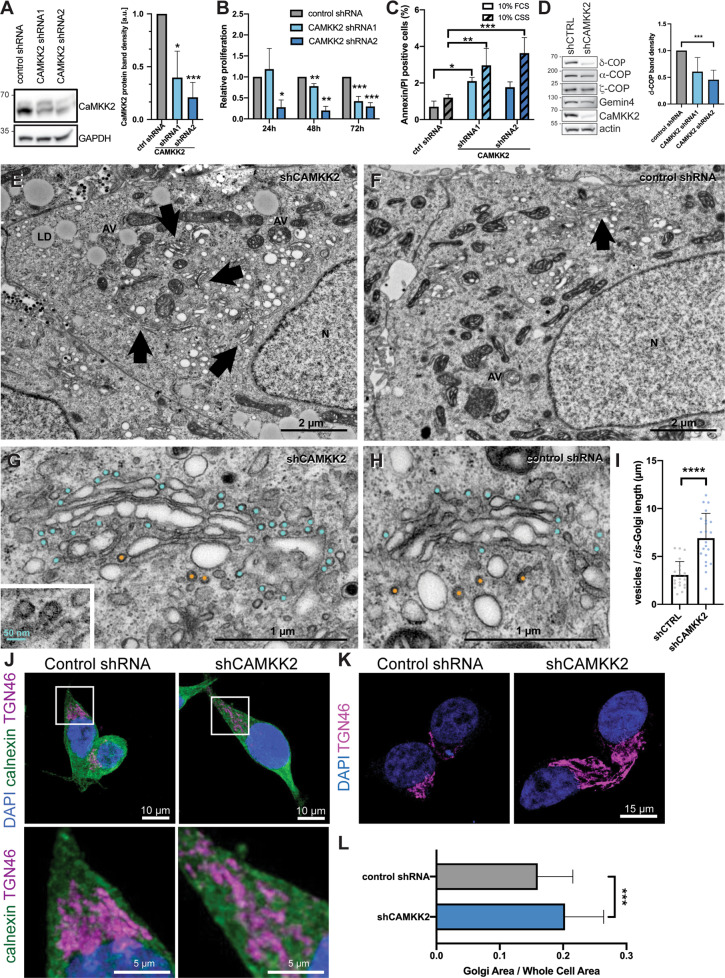
CaMKK2 regulates Golgi morphology. **A** Representative western blot of cell lysates from two CaMKK2 knockdown LNCaP cell lines. The efficiency of knockdown was quantified relative to GAPDH loading control. Mean ± SD; *n* = 3; *P = 0.0140; ***P = 0.0006. **B** Proliferation assay of LNCaP CaMKK2 KD in steady state conditions at 24, 48 and 72 h. Mean ± SD; *n* = 3; *P < 0.05; **P < 0.01; ***P < 0.001. **C** Percentage of Annexin V and PI-positive cells present in LNCaP KD following 72 h in 10% foetal calf serum (FCS) or 10% charcoal-stripped serum (CSS) serum assessed by flow cytometry. Mean ± SD; *N* = 3; *P < 0.0170, **P < 0.006, ***P < 0.0008. **D** Representative western blots of steady-state level COPI and Gemin4 protein levels in CaMKK2 knockdown (shRNA2) LNCaP cells. Densitometry values for δ-COP were generated in ImageJ relative to loading control. *N* = 3 for shRNA1 and *N* = 4 for shRNA2; *P* = 0.0281 for CAMKK2 shRNA1 and ***P = 0.0009 for CAMKK2 shRNA2 compared to control. **E**, **F** Transmission electron microscopic (TEM) images from CAMKK2 knockdown (shRNA2) and control LNCaP cells grown in regular media showing morphological differences in Golgi network (arrows), lipid droplets (LD) and autophagic vesicles (AV). *N* denotes nucleus. Scale bars 2 µm. **G**, **H** Electron micrographs of control and CaMKK2-depleted LNCaP cells showing the Golgi apparatus at higher magnification. Clathrin-coated membrane buds are marked in orange and non-clathrin-coated vesicles, presumably COPI coated, are marked in cyan. Inset shows COP-coated membrane buds; scale bar 50 nm. Scale bars 2 µm. **I** Quantification of vesicles per length of *cis*-Golgi membrane. Mean ± SD; *n* = 22; ****P < 0.0001. **J** Immunostaining of TGN46 (Golgi) in magenta showed a more dispersed distribution in the LNCaP CAMKK2 shRNA2 cell line compared to control. Endogenous calnexin (green) shows the endoplasmic reticulum. Scale bar = 10 µm. Boxed inlays are shown below. Scale bar = 5 µm. **K** Representative images showing maximal projections from *z*-stack confocal series of TGN46 (magenta) and DAPI (blue). **L** Quantification of the Golgi area normalised to the whole-cell area show an increase in Golgi area in CAMKK2-deficient cells. Sixty-two cells were quantified across three replicates for each cell line. Mean ± SD; ***P = 0.0004.

To assess the impact of CaMKK2 on membrane trafficking and organelle structure, we generated a stable knockdown of *CAMKK2* in a prostate cancer cell line, LNCaP, using two independent shRNA constructs (Fig. 3A). This significantly reduced proliferation (Fig. 3B). Reduced CaMKK2 expression did not induce cell death under normal growth conditions but did induce cell death when cells were cultured in conditions of androgen deprivation in the presence of CSS (Fig. 3C) [30]. The magnitude of the impact on cell proliferation and induction of cell death correlated with the efficiency of knockdown for the two independent shRNAs. Knocking down CAMKK2 expression also reduced the steady-state levels of Gemin4 and COPI coatomer (α-COP and ζ-COP), but most significantly, δ-COP expression was reduced **(**Fig. 3D). We therefore hypothesised that if these interactions were functionally important then knocking down CAMKK2 expression would lead to cellular phenotypes comparable to those arising from reducing COPI coatomer subunit expression. Knockdown of other COPI components such as *COPZ1* has been reported to cause Golgi apparatus collapse, blockage of autophagy, and induce apoptosis [31].

To assess these effects in our CaMKK2 knockdown and control cells, we performed electron microscopy. A striking phenotype in the CaMKK2 knockdown cells was an increase in the area occupied by the Golgi (marked by arrows) compared to the control (Fig. 3E, F). Due to the high resolution of the electron micrographs, we were able to observe that the number of small membrane vesicles and buds, primarily on the *cis*-face of the Golgi, were increased in the knockdown cells compared to the control (Fig. 3G, H; marked in cyan), while the number of clathrin-coated pits on the *trans*-face appeared to be similar (Fig. 3G, H; marked in orange). Quantification of the number of vesicles associated with the *cis*-Golgi in these micrographs confirmed that there was a significant increase in the CAMKK2 knockdown condition versus control (Fig. 3I). In higher-magnification micrographs, vesicles budding from the *cis*-Golgi were identified as COPI-coated vesicles based on their diameter (~50 nm), spatial distribution and the presence of a coat (Fig. 3G inset). These data indicated that an inhibition of COPI retrograde trafficking from the Golgi to the ER may occur in the CaMKK2 knockdown resulting in perturbed vesicle trafficking.

To further investigate whether CaMKK2 knockdown had an impact on vesicle trafficking in the Golgi network, we immuno-stained the CaMKK2 knockdown cells with a *trans*-Golgi marker, TGN46, and compared this staining to control (Fig. 3J). While the ER marker calnexin looked similar, the *trans*-Golgi network appeared extended and more dispersed in the CaMKK2 knockdown cells. This observation was corroborated by examining TGN46 staining in projection images (Fig. 3K). Quantitation of this staining showed that CaMKK2-depleted cells had a significantly enlarged Golgi network (30% expansion) compared to the control (Fig. 3L).

### Knockdown of Gemin4 negatively impacts on cell proliferation, COPI coatomer expression and Golgi membrane homoeostasis

**Fig. 4:**
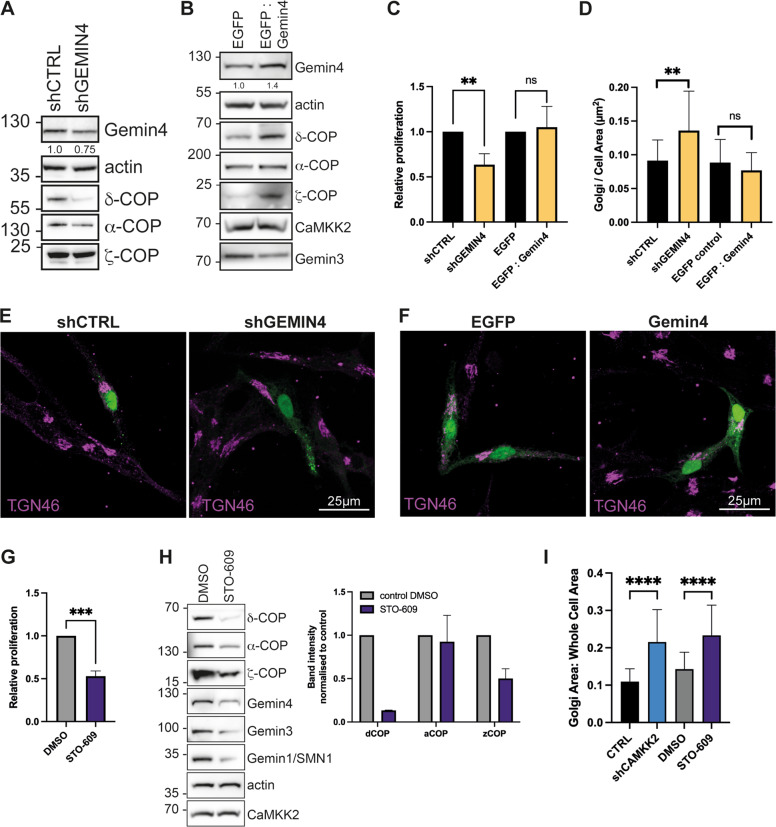
Gemin4 regulates COPI subunit expression, proliferation and Golgi morphology. **A** LNCaP cells were treated with shRNA targeting *GEMIN4* for 48 h. Protein expression levels were assessed by western blotting. Actin was used as loading control. **B** LNCaP cells were transiently transfected with a Gemin4 expression construct and protein expression levels were assessed by western blotting. Actin was used as a loading control. **C** Cell proliferation in Gemin4 knockdown and overexpressing LNCaP cells was compared to the respective control: control shRNA and EGFP. Cells were grown for 48 h in media supplemented with 10% charcoal-stripped serum. Mean ± SD, *N* = 3 independent experiments with 3 technical repeats; **P = 0.0064, ns = 0.72. **D** Quantification of Golgi area (TGN46-postive) from confocal images (shown in **E**, **F**) in Gemin4 knockdown and overexpressing LNCaP cells. Mean ± SD. Twenty-two knockdown cells and 20 overexpressing cells were analysed per group. **P = 0.0030, ns = 0.24. **E**, **F** Images showing maximum intensity *z*-projections of confocal images of LNCaP cells transiently expressing EGFP-tagged shRNA or a Gemin4 expression constructs. Cell were stained with a TGN46 antibody (magenta). Scale bar, 25 µm. **G** Bar graph showing proliferation of LNCaP cells treated with 25 µM STO-609 for 120 h normalised to the DMSO control. Three independent experiments with three technical repeats were analysed. Mean ± SD; ***P = 0.0002. **H** LNCaP cells were treated with 25 µM STO-609 for 120 h. Protein expression of COPI components and Gemins were assessed by western blotting of 25 µg cell extracts separated on a SDS-PAGE gel. Actin was used as a loading control. Densitometry was performed on two experiments and normalised to control. Mean ± SD. **I** Bar graph showing the impact of 25 µM STO-609 treatment for 120 h in LNCaP cells on Golgi area (TGN46-positive) compared to CaMKK2 knockdown (shRNA2) cells. Mean ± SD. N = 31 cells; ****P < 0.0001.

It is known that the knockdown of individual COPI subunits results in abnormal Golgi structure and inhibits proliferation. However, the impact of knocking down Gemin4 expression on these processes have not been investigated [31, 32]. Knocking down *GEMIN4* (Fig. 4A) resulted in a reduction in δ-COP expression and overexpressing Gemin4 (Fig. 4B) increased δ-COP expression. Expression levels of α-COP and ζ-COP were affected by the level of Gemin4 expression but the effect was not as strong as for δ-COP. Proliferation was significantly impaired in Gemin4 knockdown cells, while overexpression of Gemin4 had no significant impact (Fig. 4C). These results provided evidence for a significant role for Gemin4 in COPI coatomer stability. Next we investigated whether knockdown of Gemin4 resulted in an increase in Golgi area in a similar fashion to reducing CaMKK2 expression. Confocal imaging of TGN46 staining showed a significantly larger Golgi area in cells where Gemin4 expression was knocked down by shRNA (Fig. 4D, E). In summary, Gemin4 knockdown has an equivalent impact on δ-COP expression and Golgi area to reducing CaMKK2 expression.

### A CaMKK2 inhibitor increases Golgi area while reducing proliferation and COPI levels

To further investigate the relationship between CaMKK2, Gemin4 and δ-COP, we treated cells with a CaMKK2 inhibitor, STO-609 [4]. Proliferation was impaired after 120 h of treatment with STO-609 corroborating previous studies in LNCaP cells using the same inhibitor concentration ([4]; Fig. 4G). While the protein levels of CaMKK2 were unaffected by STO-609 treatment, the levels of δ-, α-, ζ-COP and Gemins were all reduced (Fig. 4H). We then assessed Golgi area as previously described and found that STO-609 treatment led to an increase in Golgi area equivalent to that observed in the CaMKK2 knockdown condition (Fig. 4I). These data further indicate that there is an important functional relationship between CaMKK2, Gemin4 and the COPI complex and the effect of STO-609 treatment implies that this requires the kinase activity of CaMKK2.

### CaMKK2 expression affects the unfolded protein response (UPR), lysosomal acidification and autophagy

**Fig. 5:**
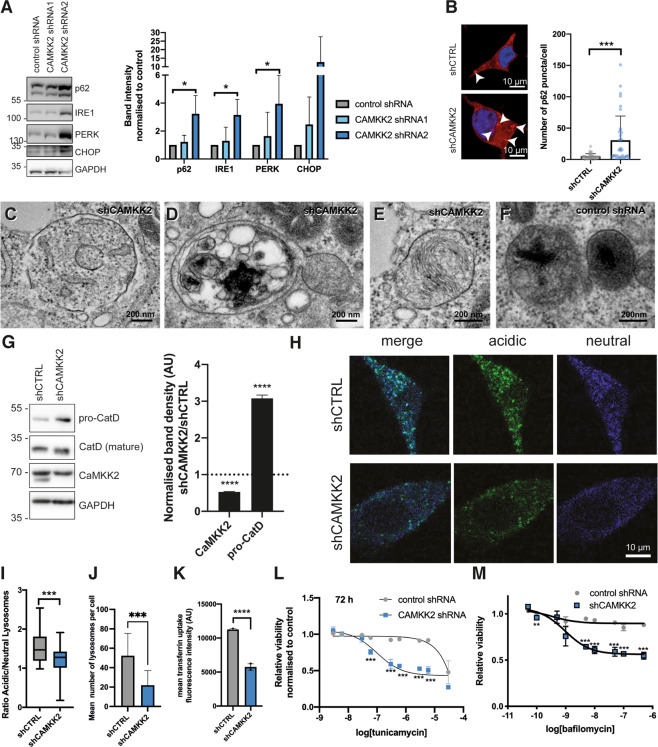
Loss of CaMKK2 results in ER stress and an increased sensitivity to a lysosomal acidification inhibitor. **A** Representative western blot of unfolded protein response (UPR) and autophagy proteins: p62, IRE1, PERK, CHOP, in CAMKK2 knockdown LNCaP cell lysates. Densitometry was normalised to loading control for each sample. *N* = 3 for IRE1 and *N* = 4 for PERK and LC3. Mean ± SD; *P < 0.05. **B** Representative confocal images of control and CAMKK2 knockdown (shRNA2) LNCaP cells stained for p62 and DAPI. The number of p62 puncta (arrow heads) were analysed in 35 cells across 3 replicates. Mean ± SD; ***P = 0.0002. Scale bar = 10 µm. **C**, **D** Representative transmission electron microscopic (TEM) images of CAMKK2 knockdown (shRNA2) LNCaP cells showing autolysosomes including hemi-fused autophagosome-lysosome structures. Scale bars 200 nm. **E**, **F** Representative TEM images showing the lysosome morphology in CAMKK2 knockdown (shRNA2) and control shRNA cells. Scale bars 200 nm. **G** Quantification of uncleaved pro-Cathepsin D in cell lysates from control and CAMKK2 knockdown (shRNA2) cells by western blotting. Graph displays values from shCAMKK2 normalised to the control knockdown, mean ± SD, *N* = 3, *P* < 0.0001. **H** Representative multiphoton live cell images of LNCaP cells stably expressing control or CAMKK2-targeting shRNA and stained with Lysosensor DND-160. Scale bar 10 µm (for all images). **I** Quantification showing the ratio of acidic:neutral lysosomes using CTFC values for blue/green channels. Thirty-eight cells were analysed across three replicates. The box and whisker plot show 5–95 percentile with the mean indicated with a line; ***P = 0.0002. **J** The mean number of lysosomes per cell were quantified from the Lysosensor DND-160 data. Mean ± SD for 15 cells in three replicates; ***P = 0.0002. **K** Uptake of Alexa647-transferrin for 10 min was measured to investigate the endocytosis of the transferrin receptor. Samples were analysed by a FACS assay showed a significant reduction compared to control in the CaMKK2 knockdown LNCaP cell line. The bar graph shows mean with SD, *N* = 3 with 3 technical repeats per experiment that each analysed the mean fluorescence of 10,000 cells. ****P < 0.0001, two-way ANOVA. **L** A viability assay showing the tunicamycin dose response at 24, 48 and 72 h in LNCaP control and CaMKK2 knockdown. Mean ± SD; *P < 0.05; **P < 0.01; ***P < 0.001. **M** A viability assay showing the response of CaMKK2 shRNA2 LNCaP cells to increasing concentrations of Bafilomycin A1 dose at 72 h normalised to control. Mean ± SD; **P < 0.01; ***P < 0.001.

ER and Golgi function play important roles in protein folding and secretion, lysosomal biogenesis and autophagy among other essential cellular processes. To assess the impact of knocking down CaMKK2 on protein folding capacity, we blotted cell lysates for components of the UPR and observed an increase in the expression of two important UPR regulators, IRE1 and PERK (Fig. 5A). In addition, the levels of p62, a receptor required for cargo-mediated autophagy, were elevated upon CAMKK2 knockdown in cell lysates and we observed an increase in p62 punctae in confocal images (Fig. 5A, B). Since autophagy is a dynamic process, elevated levels of p62 may either reflect increased autophagic flux or abortive autophagy. To gain more insights, we performed electron microscopy on the CaMKK2 knockdown and control cells. We observed a twofold increase of autophagosomes and autolysosomes in the knockdown cells (Fig. 5C; *P < 0.05, *n* = 20), and this included a hemi-fused autophagosome with a lysosome indicating a defect in autophagosome degradation by lysosomes (Fig. 5D). This strongly suggested that the increase in p62 expression and punctae reflected impaired autophagy. Electron microscopy also revealed that many lysosomes in the knockdown cells were not as electron dense as normal lysosomes, potentially reflecting defects in lysosomal function (Fig. 5E, F).

Cathepsin D is a lysosomal enzyme that needs to be glycosylated within Golgi and ultimately activated by proteolytic cleavage upon trafficking to the lysosome. Cathepsin D processing is assessed based on the molecular weights of uncleaved and mature forms of the enzyme. Using western blotting of cell lysates, we found that the CaMKK2 knockdown cells compared to control had threefold higher uncleaved pro-Cathepsin D (Fig. 5G), implying either perturbations in Golgi processing of Cathepsin D and transport to lysosomes or impaired lysosomal acidification. Lysosensor, a ratiometric dye that localises to acidic vesicles (mainly lysosomes), was used to assess this further. The data showed a decrease in the fluorescence in LNCaP CaMKK2 knockdown cells compared to control, which indicated a lysosomal acidification defect (Fig. 5H, I and Supplementary Fig. 2). In addition, the number of lysosomes per cell that were stained by the Lysosensor dye was significantly lower in CaMKK2 knockdown cells (Fig. 5J). Lysosomal activity is also required to degrade endocytosed cargo internalised at the cell surface. By starving cells and then feeding them with fluorescently labelled transferrin, we were able to quantify transferrin uptake using flow cytometry. This revealed a defect in endocytosis in the knockdown cells of a magnitude equivalent to that reported for downregulating the expression of endocytic proteins themselves or β’-COP (Fig. 5K; [25, 33]). Given the elevated levels of proteins associated with the UPR and impaired lysosomal function we hypothesised that CAMKK2 knockdown cells might be more sensitive to tunicamycin. Tunicamycin induces ER stress by inhibiting N-linked glycosylation, resulting in an accumulation of unfolded proteins in the ER. CaMKK2 knockdown cells were more than three orders of magnitude more sensitive to tunicamycin than control cells, resulting in a sub-micromolar IC50 value in a cell viability assay (Fig. 5L). Pharmacologically lysosomal acidification can also be inhibited by Bafilomycin A1 treatment. Given that lysosomal acidification was impaired by CAMKK2 knockdown, we hypothesised that Bafilomycin A1 treatment would be more cytotoxic in these cells. Treatment in a low micro-molar to nano-molar concentration range led to significant reductions in cell viability in the knockdown cells with no effects on the control line (Fig. 5M).

### An increase in Golgi area due to loss of CaMKK2 can be rescued by re-expression of CaMKK2 or Gemin4, which causes an increase in COPI coatomer expression

**Fig. 6:**
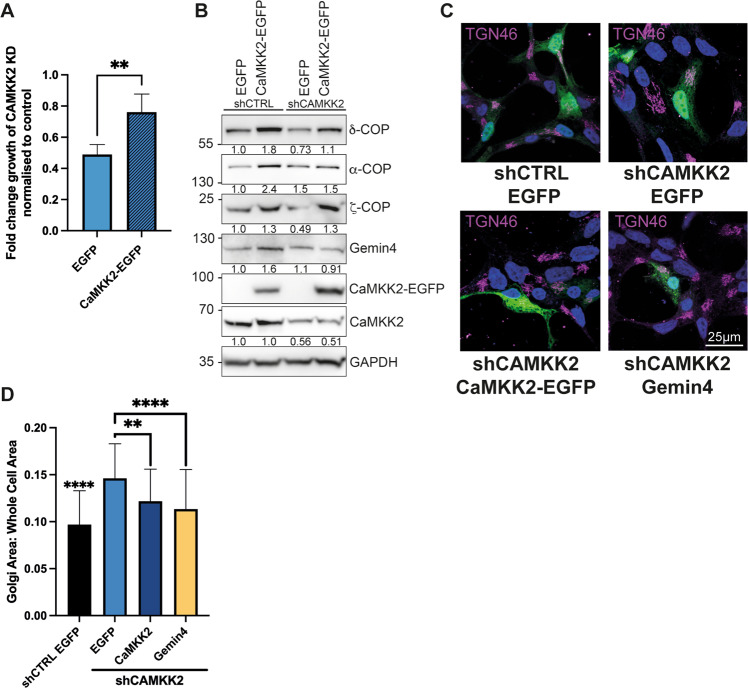
CaMKK2 expression regulates COPI coatomer expression and its re-expression can rescue the growth defect and Golgi expansion observed in CAMKK2 knockdown cells. **A** LNCaP cells stably expressing control or CAMKK2-targeting shRNA were transfected with EGFP or CaMKK2-EGFP and cultured for 96 h. The growth of the *CAMKK2* knockdown cells were normalised to control and the fold change to the seeding density was plotted. Mean ± SD; *N* = 5 with three technical repeats; **P = 0.0017. **B** Control and CAMKK2 knockdown LNCaP cells were transiently transfected with control EGFP or CaMKK2-EGFP for 48 h, cells were harvested and cell extracts were subjected to western blots for COPI coat proteins. **C** Confocal images of control and *CAMKK2* knockdown LNCaP cells transiently transfected with EGFP or CaMKK2-EGFP for 48 h, stained with TGN46 antibody and DAPI (nucleus). Scale bar = 25 µm (for all images). **D** Quantification of Golgi (TGN46-positive) area normalised to cell area of samples shown in **C**. The EGFP-control transfected CAMKK2 knockdown cells had a significantly (*P* < 0.0001) larger Golgi area compared to the control. Mean ± SEM; ****P < 0.0001, **P = 0.0017. Two independent experiments with a minimum of 20 cells per group were analysed.

To further confirm the role of CaMKK2 in Golgi vesicle trafficking, it was important to attempt to rescue the phenotypes caused by knockdown. Reduced cell proliferation upon the knockdown CaMKK2 expression was rescued by transfecting cells with a CaMKK2 overexpression construct (Fig. 6A). Transfecting CaMKK2 knockdown cells with a CaMKK2 expression construct resulted in increased protein levels of δ- and ζ-COP compared to empty vector-transfected cells (Fig. 6B). Overexpressing CaMKK2 in a control shRNA-treated cell line led to increases in δ-, α-, ζ-COP and Gemin4 (Fig. 6B). In our earlier experiments, we showed that knocking down either CaMKK2 or Gemin4 led to an increase in Golgi area. To assess their functional equivalence, we therefore attempted to revert this phenotype by overexpressing either CaMKK2 or Gemin4 against a CAMKK2 knockdown background. The overexpression of either CaMKK2 or Gemin4 led to a partial reversion of Golgi area when compared to a control shRNA-treated line transfected with an empty vector (Fig. 6C, D). The extent of the reversion was similar with either CaMKK2 or Gemin4.

## Discussion

We and others have shown that CAMKK2 is an important regulator of prostate cancer growth and plays a pro-tumorigenic role in a number of other cancer types. While there is a literature which indicates that this is due to its role in phosphorylating AMPK and regulating glycolysis and mammalian target of rapamycin (mTOR) activity, AMPK activators are not able to rescue phenotypes arising from the genetic targeting of CAMKK2 [6]. Our study has identified a novel interaction between CAMKK2 and Gemin4 and suggests that CaMKK2 has wide-ranging effects on intracellular trafficking (Fig. 1). While we were undertaking this work, a paper reported the presence of a prostate cancer risk single-nucleotide polymorphism, rs684232, that suppresses AR-dependent expression of GEMIN4 [34]. Reduced GEMIN4 expression is associated with shorter clinical survival in prostate cancer patients, and knockdown of GEMIN4 is associated with reduced cell proliferation and colony formation [26, 34]. AR activity sustains CAMKK2 expression and this supports prostate cancer cell proliferation. There is therefore an interesting inverse relationship between the effect of GEMIN4 and CAMKK2 on cancer cell proliferation, as well as in the impact of androgen on their expression. Gemin4 exists in a number of multi-protein complexes and has variously been described in the SMN complex associated with snRNP biogenesis, as an interactor with Argonaute/AGO2 in an autophagy complex and as a component of the COPI/coatomer complex interactome [29, 35]. The interactors of the CBM in Gemin4 did not consist of components of the SMN complex but rather of the COPI/coatomer complex suggesting that this is of greater functional relevance in understanding the interplay between CAMKK2 and Gemin4 (Fig. 2). In support of this, knocking down CAMKK2 or Gemin4 expression reduced the levels of the delta subunit of the COPI coatomer complex and perturbed ER–Golgi homoeostasis (Figs. 3 and 4). This suggests that CAMKK2 supports cancer cell proliferation by maintaining the membrane trafficking capacity of cells, including lysosomal acidification and activity.

Membrane trafficking between the Golgi and the ER is fundamental for maintaining organelle homoeostasis, protein synthesis and secretory capacity and consequently cell proliferation. Changes in the relative abundance of membrane in these two important organelles may therefore also underpin the reduction in cell proliferation observed previously [15, 27, 36]. Importantly, *ARCN1* (δ-COP) knockdown has previously been shown to significantly impair the viability of cancer cell lines by potentiating activation of the UPR and inhibiting phosphatidylinositol 3-kinase (PI3-kinase)/Akt signalling [36]. *ARCN1* has also been identified in genetic screens wherein knockdown impaired the growth of a panel of cancer cell lines as well as act as an autophagy modulator [32, 36]. In addition, knockdown of other COPI components such as *COPZ1* has been reported to cause Golgi apparatus collapse, blockage of autophagy and induce apoptosis [31]. In a chemical and genetic screen to identify biological targets that support tumorigenesis in non-small cell lung cancer (NSCLC), COPI coatomer subunits were identified as being required for the growth of NSCLC cells carrying mutations in KRas and LKB1 [15]. The lysosomal and membrane trafficking defects that we have observed arising from CAMKK2 knockdown closely resemble those reported in studies targeting COPI coatomer subunits (Fig. 5). Loss of COPI subunits result in impaired maturation of early endosomes, reduced transferrin endocytosis, reduced maturation of lysosomes, perturbed maturation of autophagosomes, accumulation of p62 and increased lipidation of LC3 [33]. Interestingly, we found that inhibition of the CaMKK2 kinase activity with STO-609 had a similar effect on proliferation, COPI coatomer stability and Golgi homoeostasis as knockdown of protein expression (Fig. 4G–I). Further studies are required to determine whether this is due to phosphorylations or the conformational changes occurring in the kinase domain known to regulate protein interactions [37]. Given the interest in developing CAMKK2 inhibitors for the treatment of diseases including prostate cancer, these findings also have implications for combination therapy and for future studies focussing on molecular cancer subtypes. For example, impaired lysosomal function due to CAMKK2 knockdown would be expected to affect amino acid release and sensing by mTORC1. mTORC1/mTORC2 dual inhibitors are in clinical trials for a range of malignancies, including prostate cancer [38, 39].

PTEN mutation is an important molecular subtype of poor prognosis of prostate cancer most strongly associated with aberrant mTOR activity due to effects on PI3-kinase/Akt signalling. A recent report showed that knocking out CAMKK2 in a transgenic mouse model of prostate cancer driven by PTEN loss impairs tumorigenesis [6]. Proteomics on the prostates from these mice showed a significant downregulation in the expression of coatomer COPI subunits and regulators of protein translation. These data therefore provide further correlative evidence linking CAMKK2 expression to coatomer COPI subunit expression and suggesting that CAMKK2 may support tumorigenesis by enhancing the membrane trafficking and protein turnover capacity of cells.

In conclusion, our study has provided new insights into the CAMKK2 interactome and its contribution to sustaining cell viability and proliferation as a regulator of membrane trafficking. In so doing, it suggests that CAMKK2 regulates the capacity of the secretory pathway and lysosomal function with implications for biologically informed combination treatments and cancer subtype selection.

## Supplementary information

Supplemental Figure Legends

Supplementary Figure 1

Supplementary Figure 2

Supplementary Table S1

Supplementary Table S2

## Data Availability

The data sets generated during the current study are available from the corresponding author on reasonable request.
