# Supplementary material for: CaMKK2 facilitates Golgi-associated vesicle trafficking to sustain cancer cell proliferation
Source: Cell Death Dis. 2021 Nov 1;12(11):1040. doi: 10.1038/s41419-021-04335-x (PMC8560770; doi:10.1038/s41419-021-04335-x)
Supplement: Supplementary file 1 — Supplemental Figure Legends [file 41419_2021_4335_MOESM1_ESM.docx]

**Supplementary Figure 1. Immunoprecipitation of the Gemin proteins from LNCaP cell lysates.** Using LNCaP cell lysates in full serum conditions. Endogenous Gemin3, Gemin3 and SMN/Gemin1 were immunoprecipitated and bound protein were subjected to Western blotting for CaMKK2. Control lanes include supernatant (SN) from the IgG control and the respective antibodies. The IgG immunoprecipitation constitutes the negative control.

**Supplementary Figure 2.** **Lysosomal Acidification is Impaired by Depletion of CaMKK2.** Representative images of Lysosensor DND 160 staining of LNCaP CaMKK2 shRNA 2 and control cells. Blue staining denotes lysosomes of a neutral pH (pH>4.2) whilst green staining shows acidic lysosomes (pH<4.2). Images were collected at 40x magnification using identical settings. Scale bars 20µm and for insets 10µm.
