# Supplementary figures and images for: CaMKK2 facilitates Golgi-associated vesicle trafficking to sustain cancer cell proliferation
Source: Cell Death Dis. 2021 Nov 1;12(11):1040. doi: 10.1038/s41419-021-04335-x (PMC8560770; doi:10.1038/s41419-021-04335-x)

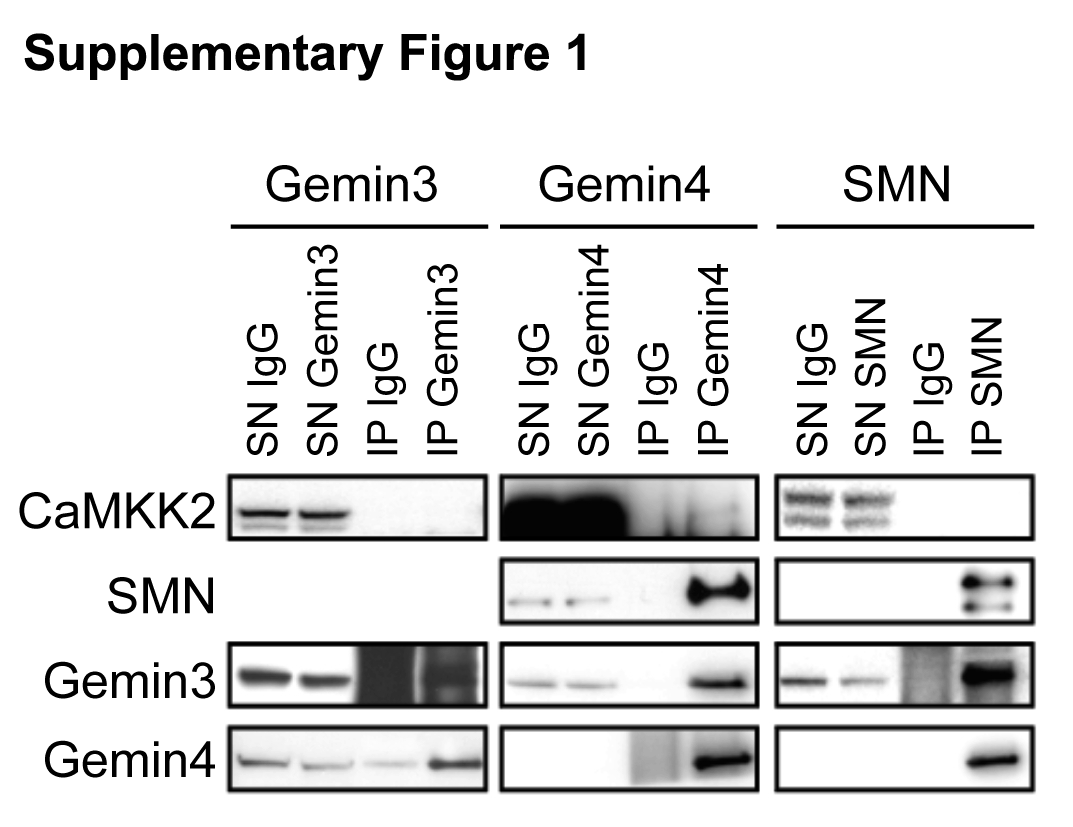

Supplement: Supplementary file 2 — Supplementary Figure 1 [file 41419_2021_4335_MOESM2_ESM.tif]

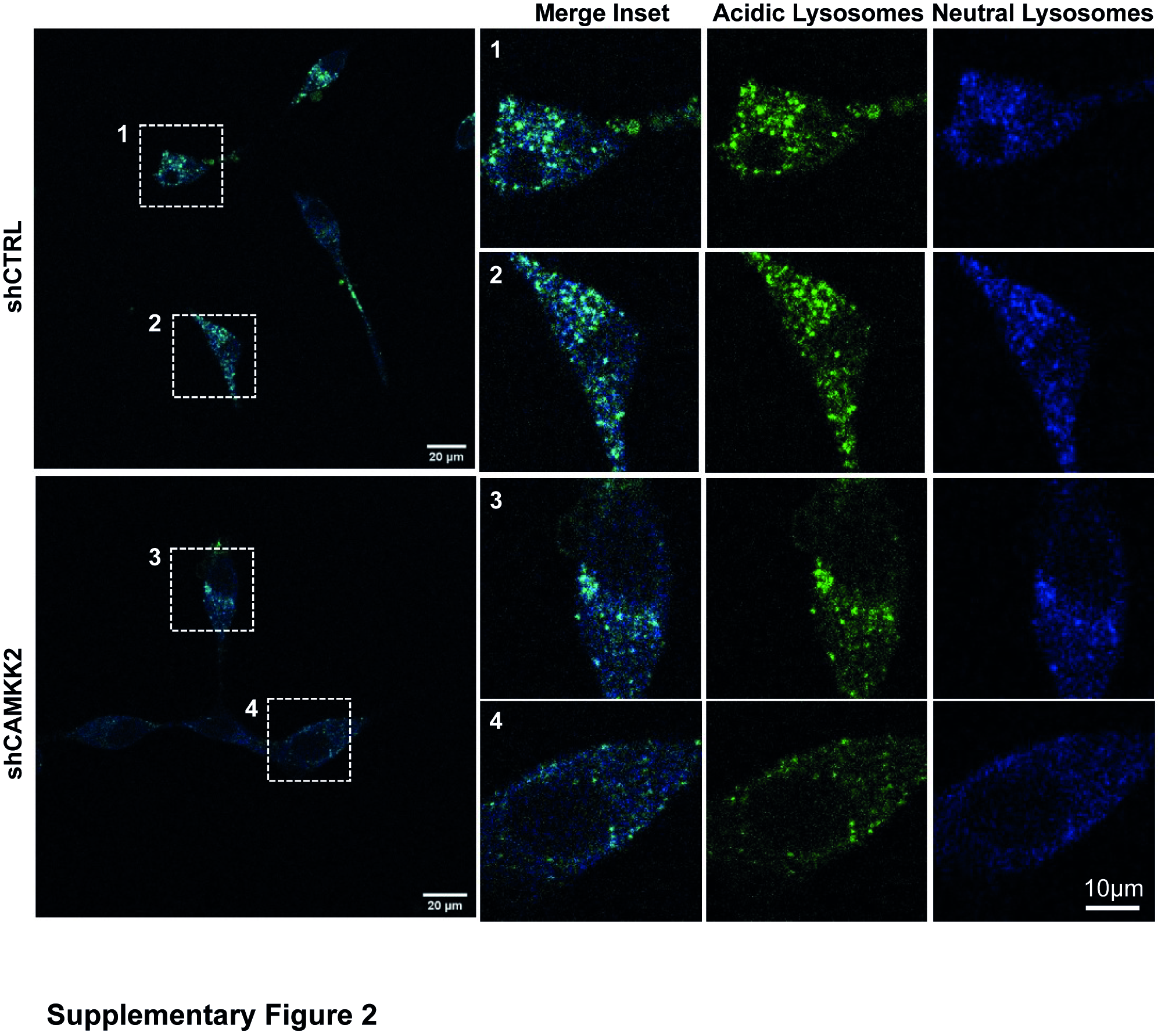

Supplement: Supplementary file 3 — Supplementary Figure 2 [file 41419_2021_4335_MOESM3_ESM.tif]
